# Prospective associations between beverage intake during the midlife and subclinical carotid atherosclerosis: The Study of Women’s Health Across the Nation

**DOI:** 10.1371/journal.pone.0219301

**Published:** 2019-07-10

**Authors:** Dongqing Wang, Carrie A. Karvonen-Gutierrez, Elizabeth A. Jackson, Michael R. Elliott, Bradley M. Appelhans, Emma Barinas-Mitchell, Lawrence F. Bielak, Ana Baylin

**Affiliations:** 1 Department of Epidemiology, School of Public Health, University of Michigan, Ann Arbor, Michigan, United States of America; 2 Division of Cardiovascular Disease, School of Medicine, University of Alabama at Birmingham, Birmingham, Alabama, United States of America; 3 Department of Biostatistics, School of Public Health, University of Michigan, Ann Arbor, Michigan, United States of America; 4 Survey Research Center, Institute for Social Research, University of Michigan, Ann Arbor, Michigan, United States of America; 5 Department of Preventive Medicine, Rush University Medical Center, Chicago, Illinois, United States of America; 6 Department of Behavioral Sciences, Rush University Medical Center, Chicago, Illinois, United States of America; 7 Department of Epidemiology, Graduate School of Public Health, University of Pittsburgh, Pittsburgh, Pennsylvania, United States of America; 8 Department of Nutritional Sciences, School of Public Health, University of Michigan, Ann Arbor, Michigan, United States of America; Universita degli Studi di Brescia, ITALY

## Abstract

**Background:**

The potential impacts of beverage intake during the midlife on future subclinical atherosclerosis among women are unclear. The aim of this study was to evaluate the prospective associations between the intakes of eight beverage groups and subclinical carotid atherosclerosis in midlife women.

**Methods:**

Data came from the Study of Women’s Health Across the Nation, a multicenter, multiethnic, and prospective cohort study. A total of 1,235 midlife women had measures of subclinical carotid atherosclerosis and repeatedly beverage intake data collected using a validated food frequency questionnaire. Beverages were aggregated into eight groups, including coffee, tea, sugar-sweetened beverages, artificially sweetened beverages, fruit juices, whole milk, milk with lower fat content, and alcoholic beverages. The associations of beverage intake with common carotid artery intima-media thickness (CCA-IMT) and adventitial diameter (CCA-AD) were estimated using linear models; the associations with carotid plaque were estimated using log-binomial models.

**Results:**

Coffee intake was associated with CCA-IMT in an inverted J-shaped pattern. After adjusting for covariates, women with >0 to <1 cup/day and 1 to <2 cups/day of coffee intake had a 0.031 mm (95% CI: 0.012, 0.051) and a 0.027 mm (95% CI: 0.005, 0.049) larger CCA-IMT, respectively, than coffee non-drinkers. Women who consumed ≥2 cups/day of coffee did not have significantly different CCA-IMT than non-drinkers. There was an inverse linear association between moderate alcoholic beverages intake and CCA-IMT (*P*-trend = 0.014). Whole milk intake had inverted U-shaped associations with CCA-IMT and carotid plaque. No significant associations were found between other beverage groups and subclinical atherosclerosis.

**Conclusions:**

In midlife women, occasional coffee intake may be associated with more subclinical atherosclerosis while moderate alcoholic beverages intake may be associated with less subclinical atherosclerosis. Future work should focus on the determination of the optimal beverage intake profile for maximum cardiovascular benefits in midlife women.

## Introduction

Cardiovascular disease (CVD) is the leading cause of mortality and morbidity among women in the United States (U.S.) [1]. About half of the U.S. women have some form of CVD, and a third of all females deaths in the U.S. are attributable to CVD [1]. Furthermore, women’s risk of CVD increases sharply after the menopause [2]. Markers of subclinical carotid atherosclerosis, including intima-media thickness, adventitial diameter, and plaque formation, are important predictors of CVD events later in life [3, 4], and can be used to quantify the cardiovascular risk in asymptomatic individuals [3–5]. Women also experience an accelerated progression of subclinical atherosclerosis during the menopausal transition [6, 7]. The steeper increase in atherosclerotic risk during the menopausal transition is likely explained by the associations between menopause and changes in cardiometabolic risk factors, including increases in total fat and visceral fat [8, 9], and elevations in total cholesterol and low-density lipoprotein (LDL) cholesterol [10]. These adverse changes, which go beyond the effects of chronological aging alone [8–10], may be preventable by lifestyle modifications [6]. Thus, the midlife in women, which is typically defined as age 40 to 65 years and includes the menopausal transition [11], represents a pivotal period for primary prevention of CVD.

Beverage intake is a major component of American people’s diets and is of high public-health importance. An average American consumes 135 gallons per year of non-water beverages [12]. Numerous compounds present in some beverages, such as caffeine [13] in coffee, and polyphenols in coffee [14–19] and tea [15, 20–22] may delay or accelerate the atherosclerotic process. Furthermore, most types of beverages consumed in the U.S. contain calories, and the increased intake of energy-dense beverages is one of the major contributors to the current obesity epidemic in America [23]. Low-calorie, artificially sweetened beverages have been proposed as a potential replacement for sugar-sweetened beverages, but there is scant evidence on the health benefits and possible adverse effects of artificially sweetened beverages [24]. Intake of alcoholic beverages is associated with clinical cardiovascular outcomes, typically in a J-shaped pattern with the lowest risk observed for light to moderate alcohol drinkers [25]. However, the potential effects of alcohol intake on subclinical atherosclerosis are unclear [26–33].

The midlife is a crucial window for cardiovascular risk prevention in women as the menopause-induced atherosclerotic risk may be counteracted by modifications in lifestyle and dietary intake during the midlife. While the potential effects of general lifestyle and overall diet quality on subclinical atherosclerosis in midlife women have recently been examined [34], few studies have examined the impacts of specific beverage intakes on subclinical atherosclerosis in this population [35–40]. In the present study, we aimed to use data from the Study of Women’s Health Across the Nation to evaluate the prospective associations between the intakes of eight non-overlapping groups of beverages and measures of subclinical carotid atherosclerosis in midlife women.

## Methods

### Study design and study population

The Study of Women’s Health Across the Nation (SWAN) is an ongoing, multicenter, multiethnic, prospective cohort study initiated in 1996 to study the natural history of the menopausal transition. Details of the SWAN protocol have been described previously [41]. Briefly, SWAN participants were recruited from seven sites across the U.S.: 1) Boston, Massachusetts; 2) Chicago, Illinois; 3) Southeastern Michigan; 4) Los Angeles, California; 5) Newark, New Jersey; 6) Pittsburgh, Pennsylvania; and 7) Oakland, California. At baseline, 3,302 women who self-identified as African American (Pittsburgh, Chicago, Detroit, and Boston), Chinese (Oakland), Japanese (Los Angeles), Hispanic (Newark), or non-Hispanic white (all sites) were enrolled. Baseline eligibility criteria included age 42 to 52 years, having an intact uterus and at least one ovary, not being pregnant or lactating, not using oral contraceptives or hormone therapy in the past three months, and having at least one menstrual cycle in the past three months. Clinic assessments began in 1996 and participants have been followed up for 15 examinations conducted approximately annually, through the most recent visit in 2015–2016. The SWAN protocols were approved by the Institutional Review Board at each site, and all participants provided written informed consent at each study visit.

**Fig 1:**
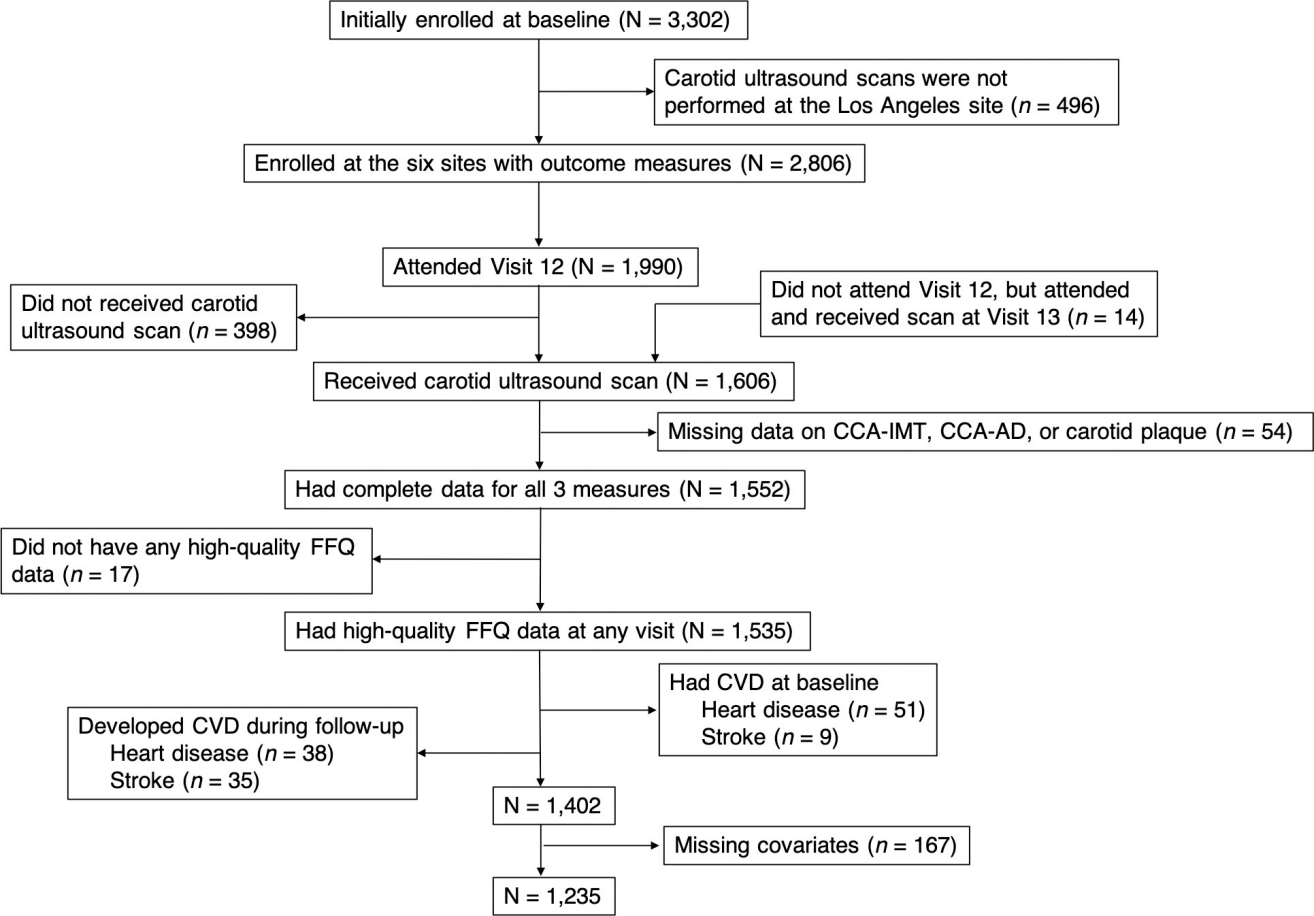
Exclusion flow of participants for the association between beverage intake and subclinical carotid atherosclerosis in the Study of Women’s Health Across the Nation (United States), 1996–2013. High-quality food frequency questionnaire data was defined as not reporting too few (< 4/day) or too many (> 16/day) solid foods, not skipping more than 10 food items on the questionnaire, and not reporting total energy intake that was too low (< 2,092 kJ/day or 500 kcal/day) or too high (>20,920 kJ/day or 5,000 kcal/day). Abbreviations: AD, adventitial diameter; CCA, common carotid artery; CVD; cardiovascular disease; FFQ, food frequency questionnaire; IMT, intima-media thickness.

Carotid ultrasound scans were performed at six sites (all sites except the Los Angeles site) at SWAN follow-up Visit 12 (2009–2011) or Visit 13 (2011–2013), with the vast majority of scans (96.7%) conducted at Visit 12. Among the 2,806 women initially enrolled at the six sites, 1,990 (70.9%) participants attended Visit 12, of whom 1,592 (80.0%) had a carotid scan at Visit 12 or Visit 13. Additionally, 14 women did not attend Visit 12, but attended and received the carotid scan at Visit 13. Thus, a total of 1,606 women had a carotid scan. From these 1,606 participants, we further excluded women who did not have all three specific measures of carotid atherosclerosis (*n* = 54); who did not have high-quality dietary data at any visit (defined as not reporting too few [< 4/day] or too many [> 16/day] solid foods, not skipping more than 10 food items on the questionnaire, and not reporting total energy intake that was too low [< 2,092 kJ/day or 500 kcal/day] or too high [>20,920 kJ/day or 5,000 kcal/day]) (*n* = 17) [34, 42]; who self-reported having heart disease (*n* = 51) or stroke (*n* = 9) at baseline or developed heart disease (*n* = 38) or stroke (*n* = 35) during the follow-up before their carotid scans; and who had missing data for the major covariates (*n* = 167). After these exclusions, the final sample study population consisted of 1,235 women (Fig 1).

### Assessment of exposures

Dietary data were collected at baseline (1996–1997), Visit 5 (2001–2003), and Visit 9 (2005–2007), except at the New Jersey site, which had dietary data at baseline and Visit 5 but not at Visit 9. Diet was measured using a modification of the 1995 version of the Block food frequency questionnaire (FFQ), which has previously been validated against dietary records and 24-hour recalls [43, 44]. Briefly, among women, the correlation coefficients between the Block FFQ and 24-hour recalls for total energy intake, protein, carbohydrate, total fat, saturated fat, monounsaturated fat, and polyunsaturated fat were 0.45, 0.53, 0.66, 0.67, 0.65, 0.60, and 0.48, respectively [44]. The FFQ included 103 food items, including 20 beverage items and 83 solid food items. Trained personnel administered the FFQ, and the participants were asked how often, on average, they consumed each item during the past year, as well as the usual portion size for each. Up to nine predefined frequencies of intake, ranging from never to ≥ 5 times/d, and three predefined portion sizes, ranging from small to large, were available for each beverage item. For the solid foods, the predefined frequencies ranged from never to ≥ 2 times/d. Total energy intake and nutrients intake were computed by multiplying the reported frequency, the reported portion size, and the corresponding nutrient content. The nutrient content was obtained from the U.S. Department of Agriculture nutrient database for standard reference, *Bowes and Church’s Food Values of Portions Commonly Used* [45], commercial food manufacturers’ websites, and food labels [46]. We computed the intakes of the 20 beverages by multiplying the reported frequency by the reported portion size, to obtain the amount of beverages intake in milliliter per day. Beverage item soy milk was not considered in the analysis due to low intake and incompatibility with other beverage items. We aggregated the remaining 19 beverages into eight non-overlapping groups, including coffee, tea, sugar-sweetened beverages (SSB), artificially sweetened beverages (ASB), fruit juices, whole milk, milk with lower fat content (2% milk, 1% milk, and skim milk), and alcoholic beverages (S1 Table). The intake of each group was calculated by summing the individual items in that group. Finally, to capture the long-term intakes, we calculated the intake of each beverage group by averaging across up to three available dietary measurements (baseline, Visit 5, and Visit 9).

### Assessment of outcomes

Centrally trained and certified sonographers obtained carotid ultrasound images at Visit 12 or Visit 13 using a Terason t3000 Ultrasound System (Teratech Corp, Burlington, MA) equipped with a variable frequency (5–12 MHz) linear array transducer [47]. Two digitized images were obtained for each of the left and right distal common carotid artery (CCA). From each of these four images, near and far wall intima-media thickness (IMT) measures of the CCA were obtained by electronically tracing the lumen-intima interface and the media-adventitia interface across a 1-cm segment proximal to the carotid bulb. One measurement was generated for each pixel over the area, for a total of approximately 140 measures for each segment. The average and maximal values for these measures were recorded for all four images, with the mean of the maximal readings of all four images used in the analyses. Adventitial diameter (AD) of the CCA was measured as the distance from the adventitial-medial interface on the near wall to the medial-adventitial interface on the far wall at end-diastole across the same CCA segments used for IMT measurement. The mean value of the average readings was used in the analyses. Sonographers at each site evaluated the presence and extent of plaque in each of five segments of the left and right carotid artery (distal and proximal CCA, carotid bulb, and proximal internal and external carotid arteries). A plaque was defined as a distinct area protruding into the vessel lumen that was at least 50% thicker than the adjacent IMT. For each segment, the degree of the plaque was graded between 0 (no observable plaque) to 3 (plaque obstructing ≥ 50% of the luminal diameter of the vessel). The grades from all segments of the combined left and right carotid artery were summed to create the plaque index [48]. The three outcomes of this study were the intima-media thickness of the common carotid artery (CCA-IMT), the adventitial diameter of the common carotid artery (CCA-AD), and the carotid plaque index. We treated CCA-IMT and CCA-AD as continuous variables [7, 34] and dichotomized carotid plaque index as ≥ 2 versus < 2 [49].

### Assessment of covariates

Self-reported covariates at baseline included age (continuous), race/ethnicity (African American, Hispanic, Chinese, or non-Hispanic white), education level (≤ high school, some college, or college degree/post-college), financial strain (somewhat/very hard paying for basics, or not hard paying for basics) [50], self-rated overall health (excellent/very good, good, or fair/poor), menopausal status based on self-reported menstrual bleeding patterns (dichotomized as premenopausal or early perimenopausal), smoking status (never, past, or current), and non-occupational physical activity level (continuous; assessed on five-point Likert and ordinal quantitative scales with total scores ranging from 3 to 15; higher values indicate more frequent engagement in non-occupational physical activity) [51]. We used the 2002 version of the Alternate Healthy Eating Index (AHEI) to quantify the overall quality of the diet at baseline, Visit 5, and Visit 9. Specifically, the AHEI was computed using nine dietary components including vegetables, fruit, nuts and legumes, the ratio of white to red meat, cereal fiber, *trans* fat, the ratio of polyunsaturated fatty acids to saturated fatty acids, multivitamin use, and alcohol intake. Each component contributes 0 to 10 points to the total score, except the dichotomous multivitamin intake, which contributes either 2.5 points for no long-term use or 7.5 points for long-term use. A maximum score indicates that the recommendation for that component was fully met, whereas a minimum score represents the least healthy behavior for that component. Intermediate intakes were scored proportionally between the minimum and the maximum scores. The nine components were then summed to obtain the total score, which ranged from 2.5 (worst overall diet) to 87.5 (best overall diet) [52]. Self-reported use of hormone therapy from baseline through the visit of the carotid scan was dichotomized as ever use or never use. Weight and height were measured by trained interviewers using a calibrated balance beam scale and a stadiometer, respectively, and BMI was calculated as weight in kilograms divided by squared height in meters. Blood pressure was calculated as the average of two seated measurements using a standard mercury sphygmomanometer. Blood samples were taken to measure fasting glucose, serum triglycerides, and serum high-density lipoprotein (HDL) cholesterol. Based on harmonized guidelines [53], elevated blood pressure was defined as systolic blood pressure ≥ 130 mm Hg, or diastolic blood pressure ≥ 85 mm Hg, or antihypertensive drug treatment. Elevated fasting glucose was defined as fasting glucose ≥ 100 mg/dL or drug treatment of elevated glucose. Elevated triglycerides was defined as fasting serum triglycerides ≥ 150 mg/dL. Reduced HDL cholesterol was defined as serum HDL cholesterol < 50 mg/dL.

### Statistical analysis

We collapsed the average beverage intakes into prespecified categories based on prior literature and the overall distributions in the study population to detect potential nonlinear associations. Specifically, we divided coffee intake into none, > 0 to < 1 serving/d, 1 to < 2 servings/d, 2 to < 4 servings/d, and ≥ 4 servings/d. We divided tea intake into none, > 0 to < 1 serving/d, 1 to < 2 servings/d, and ≥ 2 servings/d. We divided intakes of SSB, ASB, fruit juices, milk with lower fat content, and alcoholic beverages into none, > 0 to < 0.5 serving/d, 0.5 to < 1 serving/d, and ≥ 1 serving/d. We divided whole milk intake into none, > 0 to < 0.5 serving/d, and ≥ 0.5 serving/d. We defined one serving of beverage as one medium cup (237 mL) for coffee and tea, one medium glass (237 mL) for fruit juices, whole milk, and milk with lower fat content, one medium can (355 mL) for SSB, ASB, and beer, one medium glass (148 mL) for wine, and one medium shot (44 mL) for liquor.

The selection of confounders was based on *a priori* knowledge of risk factors for atherosclerosis and the empirical exposure-covariate associations in the study population. In Model 1, we adjusted for age at the carotid scan, race/ethnicity, education level, financial strain, self-rated overall health, BMI, smoking status, non-occupational physical activity level, menopausal status, use of hormone therapy from baseline to the visit of the carotid scan, and the number of missing visits for dietary measurements. All covariates in Model 1 were the baseline values except age, hormone therapy use, and the number of missing visits for dietary measurements. In Model 2, we additionally adjusted for dietary covariates, including total energy intake, AHEI, beverage condiments (for coffee and tea only), and other beverage groups that were empirically correlated with each beverage. All dietary covariates in Model 2 were the average values across available visits of baseline, Visit 5, and Visit 9. In Model 3, we additionally adjusted for clinical or cardiovascular risk factors, including elevated blood pressure, elevated fasting glucose, elevated triglycerides, and reduced HDL cholesterol, all of which were the baseline status.

We estimated the associations of beverage intake with CCA-IMT and CCA-AD using multiple linear regression models. Graphical examinations (histograms and quantile-quantile plots) revealed that both CCA-IMT and CCA-AD had normal distributions, so no transformations were performed. We estimated the associations of beverage intake with high carotid plaque index (carotid plaque index ≥ 2) using log-binomial models. We used modified Poisson models with robust variance estimation to achieve model convergence [54]. We used the participants who did not consume the beverage as the reference group. To test for linear trends, we assigned the median intake of each category to participants in the corresponding category as a continuous variable in the models. For beverage groups that displayed a potential nonlinear association with an outcome variable, we additionally examined the shape of the relationship non-parametrically using restricted cubic splines [55]. Tests for nonlinearity and computations of *P*-curve were conducted using likelihood ratio tests comparing the model with only the linear term to the model with the linear and the cubic spline terms.

We performed three sensitivity analyses to examine the robustness of the results. First, to assess selection bias due to attrition and missing data, we used inverse probability weighting to develop a non-response weight for each retained participant based on her baseline predictors of attrition (race/ethnicity, education level, financial strain, marital status, self-rated overall health, depressive symptoms, BMI, smoking status, physical activity level, menopausal status, AHEI, elevated blood pressure, elevated fasting glucose, and reduced HDL cholesterol), and repeated the analyses using the weights. Second, as some women might abstain from a beverage before baseline due to existing health conditions or underlying health concerns, we excluded participants who did not consume the beverage (i.e., the long-term abstainers) and used the next nonzero intake category as the reference group. Third, as some women might start abstaining from a beverage during the follow-up due to health considerations, we excluded participants who stopped consuming a beverage during the follow-up (i.e., the former drinkers). Fourth, we restricted to the 792 women with beverage intake data from all three time points (baseline, Visit 5, and Visit 9). Fifth, to examine the impacts of total energy adjustment, we dropped total energy intake from the final models. All analyses were conducted using SAS 9.4 (SAS Institute Inc., Cary, NC) at a two-sided alpha level of 0.05.

## Results

We summarized the general characteristics of the sample study population in Table 1. The median age of the participants at baseline was 46.2 years with an interquartile range (IQR) of 4.1 years. Over half of the participants (52.8%) were non-Hispanic white, 28.4% were African American, 12.9% were Chinese, and 5.9% were Hispanic. At baseline, 62.3% of the women were never smokers, 25.4% were past smokers, and 12.2% were current smokers. The median BMI at baseline was 26.5 (IQR: 8.5). The mean CCA-IMT and CCA-AD at the visit of the carotid scan were 0.9 mm (standard deviation: 0.1) and 7.2 mm (standard deviation: 0.7), respectively, and 25.3% of the participants had high carotid plaque index.

**Table 1:** General characteristics and beverage intake among 1,235 participants of the Study of Women’s Health Across the Nation (United States), 1996–2013^a^.

	N = 1,235
Major covariates	
Age at baseline, year, median (IQR)	46.2 (4.1)
Age at the carotid scan, year, median (IQR)	60.0 (4.0)
Race and ethnicity, *n* (%)	
- African American	351 (28.4)
- Hispanic	73 (5.9)
- Chinese	159 (12.9)
- Non-Hispanic white	652 (52.8)
Education level, *n* (%)	
- High school or less	259 (21.0)
- Some college	377 (30.5)
- College degree/post-college	599 (48.5)
Somewhat/very hard to pay for basics, *n* (%)	392 (31.7)
Self-rated overall health, *n* (%)	
- Excellent/very good	787 (63.7)
- Good	337 (27.3)
- Fair/poor	111 (9.0)
Total energy intake^b^, kJ/d, median (IQR)	6885.6 (2980.3)
BMI, median (IQR)	26.5 (8.5)
Smoking status, *n* (%)	
- Never	770 (62.4)
- Past	314 (25.4)
- Current	151 (12.2)
Non-occupational physical activity score, mean (SD)	7.8 (1.8)
Alternate Healthy Eating Index^b^, mean (SD)	37.2 (9.2)
Menopausal status, *n* (%)	
- Early perimenopausal	548 (44.4)
- Premenopausal	687 (55.6)
Hormone therapy use^c^, *n* (%)	531 (43.0)
Elevated blood pressure, *n* (%)	326 (26.4)
Elevated fasting glucose, *n* (%)	260 (21.1)
Elevated triglycerides, *n* (%)	216 (17.5)
Reduced HDL cholesterol, *n* (%)	409 (33.1)
Number of missing dietary measurements, *n* (%)	
- 0	792 (64.1)
- 1	314 (25.4)
- 2	129 (10.5)
Beverage intake^d^	
Coffee drinkers, *n* (%)	925 (74.9)
- Intake of coffee, cups/d, median (IQR)	1.3 (1.5)
Tea drinkers, *n* (%)	961 (77.8)
- Intake of tea, cups/d, median (IQR)	0.4 (1.1)
Sugar-sweetened beverages drinkers, *n* (%)	1008 (81.6)
- Intake of SSB, cans/d, median (IQR))	0.3 (0.8)
Artificially sweetened beverages drinkers, *n* (%)	648 (52.5)
- Intake of ASB, cans/d, median (IQR)	0.3 (0.7)
Fruit juices drinkers, *n* (%)	1065 (86.2)
- Intake of fruit juices, glasses/d, median (IQR)	0.4 (0.6)
Whole milk drinkers, *n* (%)	236 (19.1)
- Intake of whole milk, glasses/d, median (IQR)	0.3 (0.6)
Milk with lower fat content drinkers, *n* (%)	1080 (87.5)
- Intake of milk with lower fat content, glasses/d, median (IQR)	0.5 (0.9)
Alcoholic beverages drinkers, *n* (%)	763 (61.8)
- Intake of alcoholic beverages, servings/d, median (IQR)	0.3 (0.7)
Subclinical atherosclerosis^e^	
CCA-IMT, mm, mean (SD)	0.9 (0.1)
CCA-AD, mm, mean (SD)	7.2 (0.7)
High carotid plaque index, *n* (%)	312 (25.3)

Abbreviations: AD, adventitial diameter; ASB, artificially sweetened beverages; CCA, common carotid artery; IMT, intima-media thickness; IQR, interquartile range; SD, standard deviation; SSB, sugar-sweetened beverages.

^a^ Values are means (standard deviations) for continuous variables with normal distributions, medians (interquartile ranges) for continuous variables with skewed distributions, and counts (percentages) for categorical variables. Percentages of polytomous variables may not sum to 100% due to rounding. The variables are the baseline measures unless specified otherwise.

^b^ Averaged across available visits of baseline (1996–1997), Visit 5 (2001–2003), and Visit 9 (2005–2007).

^c^ Defined as reported use of hormone therapy at any time from baseline to the visit of the carotid scan.

^d^ Values are the averages across available visits of baseline, Visit 5, and Visit 9. One serving was defined as one medium cup (237 mL) for coffee and tea, one medium glass (237 mL) for fruit juices, whole milk, and milk with lower fat content, one medium can (355 mL) for sugar-sweetened beverages, artificially sweetened beverages, and beer, one medium glass (148 mL) for wine, and one medium shot (44 mL) for liquor. The intakes were calculated among the drinkers only.

^e^ Measured either at Visit 12 (2009–2011) or Visit 13 (2011–2013).

The stability of beverage intake over time was fair to good, with fruit juices being the least stable (Spearman *r* = 0.38 between baseline and Visit 5 and 0.33 between baseline and Visit 9), and coffee (Spearman *r* = 0.74 between baseline and Visit 5 and 0.73 between baseline and Visit 9) and alcoholic beverages being the most stable (Spearman *r* = 0.77 between baseline and Visit 5 and 0.72 between baseline and Visit 9). There were weak correlations between coffee and tea (Spearman *r* = -0.17), coffee and alcoholic beverages (Spearman *r* = 0.29), SSB and fruit juices (Spearman *r* = 0.17), ASB and milk with lower fat content (Spearman *r* = 0.13), and whole milk and milk with lower fat content (Spearman *r* = -0.21).

**Fig 2:**
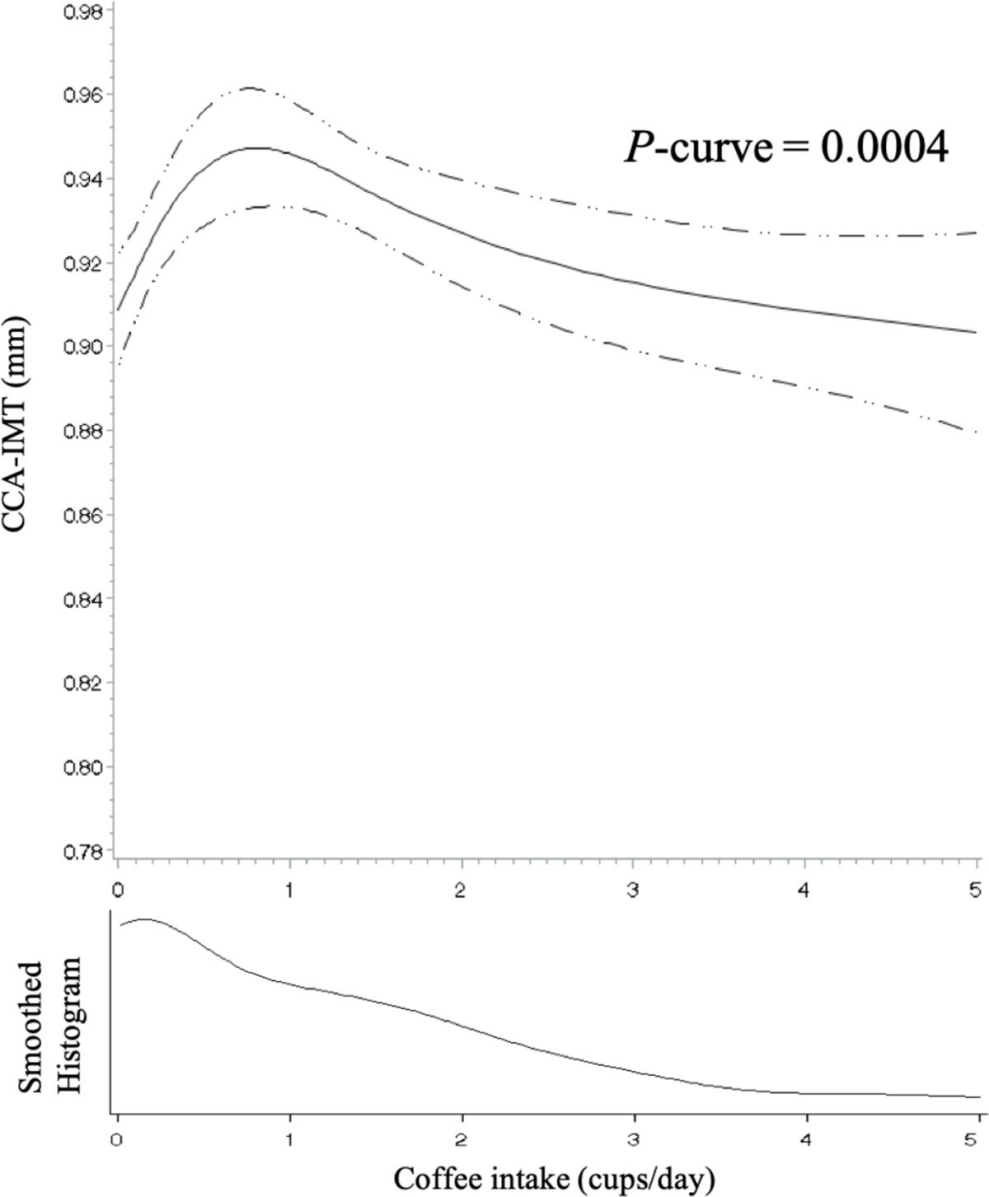
Association between coffee intake and common carotid artery intima-media thickness among 1,235 participants of the Study of Women’s Health Across the Nation (United States) using restricted cubic splines, 1996–2013. The solid line represents the predicted least squares means computed using sample means for continuous covariates and sample percentages for categorical covariates. The dashed lines represent the 95% confidence limits. Four knots were placed at 5th, 35th, 65th, and 95th percentiles of the sample distribution corresponding to 0, 0.29, 1.40, and 4.35 cups/d, respectively. One serving of coffee was defined as one medium cup (237 mL). *P*-curve was computed using the likelihood ratio test comparing the model with only the linear term to the model with the linear and the cubic spline terms. The model was adjusted for age at the carotid scan, race/ethnicity, education level, financial strain, self-rated overall health, BMI, smoking status, non-occupational physical activity level, menopausal status, use of hormone therapy from baseline to the visit of the carotid scan, the number of missing visits for dietary measurements, total energy intake, Alternate Healthy Eating Index, intake of tea, intake of alcoholic beverages, intake of beverage condiments, elevated blood pressure, elevated fasting glucose, elevated triglycerides, and reduced HDL cholesterol. Extreme coffee intake (> 5 cups/d) was trimmed from the graph due to sparse data. Abbreviations: CCA, common carotid artery; IMT, intima-media thickness.

We detected an inverted J-shaped association between coffee intake and CCA-IMT (Table 2). After fully adjusting for covariates, women who consumed > 0 to < 1 cup/d and 1 to < 2 cups/d of coffee had a 0.031 mm (95% CI: 0.012, 0.051) and a 0.027 mm (95% CI: 0.005, 0.049) larger CCA-IMT, respectively, than coffee non-drinkers. Women who consumed 2 to < 4 cups/d or ≥ 4 cups/d of coffee did not have significantly different CCA-IMT than non-drinkers. Restricted cubic spline regression also supported the nonlinear association between coffee intake and CCA-IMT (*P*-curve = 0.0004) (Fig 2). We did not find significant associations between coffee intake and CCA-AD or carotid plaque after fully adjusting for covariates.

**Table 2:** Average coffee intake and subclinical carotid atherosclerosis among 1,235 participants of the Study of Women’s Health Across the Nation (United States), 1996–2013^a^.

Coffee, servings/d						*P*-trend^b^
	None	> 0 to < 1	1 to < 2	2 to < 4	≥ 4	
*n* (%)	310 (25.1)	355 (28.7)	273 (22.1)	223 (18.1)	74 (6.0)	
CCA-IMT, mm						
Model 1^c^	*Ref*	0.027 (0.008, 0.046)^g^	0.021 (-0.000, 0.042)	0.010 (-0.013, 0.032)	-0.006 (-0.039, 0.028)	0.49
Model 2^d^	*Ref*	0.029 (0.010, 0.049)^g^	0.025 (0.003, 0.047)^f^	0.016 (-0.008, 0.040)	0.001 (-0.036, 0.039)	0.83
Model 3^e^	*Ref*	0.031 (0.012, 0.051)^g^	0.027 (0.005, 0.049)^f^	0.018 (-0.006, 0.042)	0.005 (-0.033, 0.043)	0.96
CCA-AD, mm						
Model 1^c^	*Ref*	0.01 (-0.08, 0.10)	-0.05 (-0.15, 0.05)	0.07 (-0.04, 0.18)	-0.09 (-0.25, 0.07)	0.78
Model 2^d^	*Ref*	0.01 (-0.09, 0.10)	-0.05 (-0.16, 0.05)	0.06 (-0.05, 0.18)	-0.11 (-0.29, 0.06)	0.64
Model 3^e^	*Ref*	0.01 (-0.08, 0.11)	-0.04 (-0.15, 0.06)	0.07 (-0.05, 0.18)	-0.09 (-0.27, 0.09)	0.79
Carotid plaque						
Model 1^c^	*Ref*	1.09 (0.82, 1.43)	1.21 (0.90, 1.61)	1.42 (1.06, 1.90)^f^	1.11 (0.73, 1.67)	0.16
Model 2^d^	*Ref*	1.06 (0.80, 1.41)	1.16 (0.86, 1.57)	1.30 (0.95, 1.78)	1.00 (0.64, 1.57)	0.52
Model 3^e^	*Ref*	1.08 (0.82, 1.43)	1.20 (0.89, 1.62)	1.36 (0.99, 1.85)	1.02 (0.65, 1.61)	0.42

Abbreviations: AD, adventitial diameter; CCA, common carotid artery; IMT, intima-media thickness.

^a^ Values for CCA-IMT/CCA-AD are mean differences (95% CIs) from linear models. Values for carotid plaque are risk ratios (95% CIs) of high carotid plaque index (≥ 2) from log-binomial models. Modified Poisson models with robust variance estimation were used to handle model convergence issues. One serving of coffee was defined as one medium cup (237 mL).

^b^ Computed by assigning the median intake of each category to participants in the corresponding category as a continuous variable.

^c^ Adjusted for age at the carotid scan (continuous), race/ethnicity (African American, Hispanic, Chinese, or non-Hispanic white), education level (≤ high school, some college, or college degree/post-college), financial strain (somewhat/very hard paying for basics, or not hard paying for basics), self-rated overall health (excellent/very good, good, or fair/poor), BMI (continuous), smoking status (never, past, or current), non-occupational physical activity level (continuous), menopausal status (premenopausal or early perimenopausal), use of hormone therapy from baseline to the visit of the carotid scan (ever or never), and the number of missing visits for dietary measurements (0, 1, or 2). The baseline covariates were used unless specified otherwise.

^d^ Model 1 + dietary covariates: The dietary covariates included total energy intake, Alternate Healthy Eating Index, intake of tea, intake of alcoholic beverages, and intake of beverage condiments, all of which were continuous and the average values across available visits of baseline, Visit 5, and Visit 9.

^e^ Model 2 + cardiovascular risk factors: The cardiovascular risk factors included elevated blood pressure, elevated fasting glucose, elevated triglycerides, and reduced HDL cholesterol, all binary and measured at baseline.

^f^ *P*<0.05 (compared to the reference group).

^g^ *P*<0.01 (compared to the reference group).

**Fig 3:**
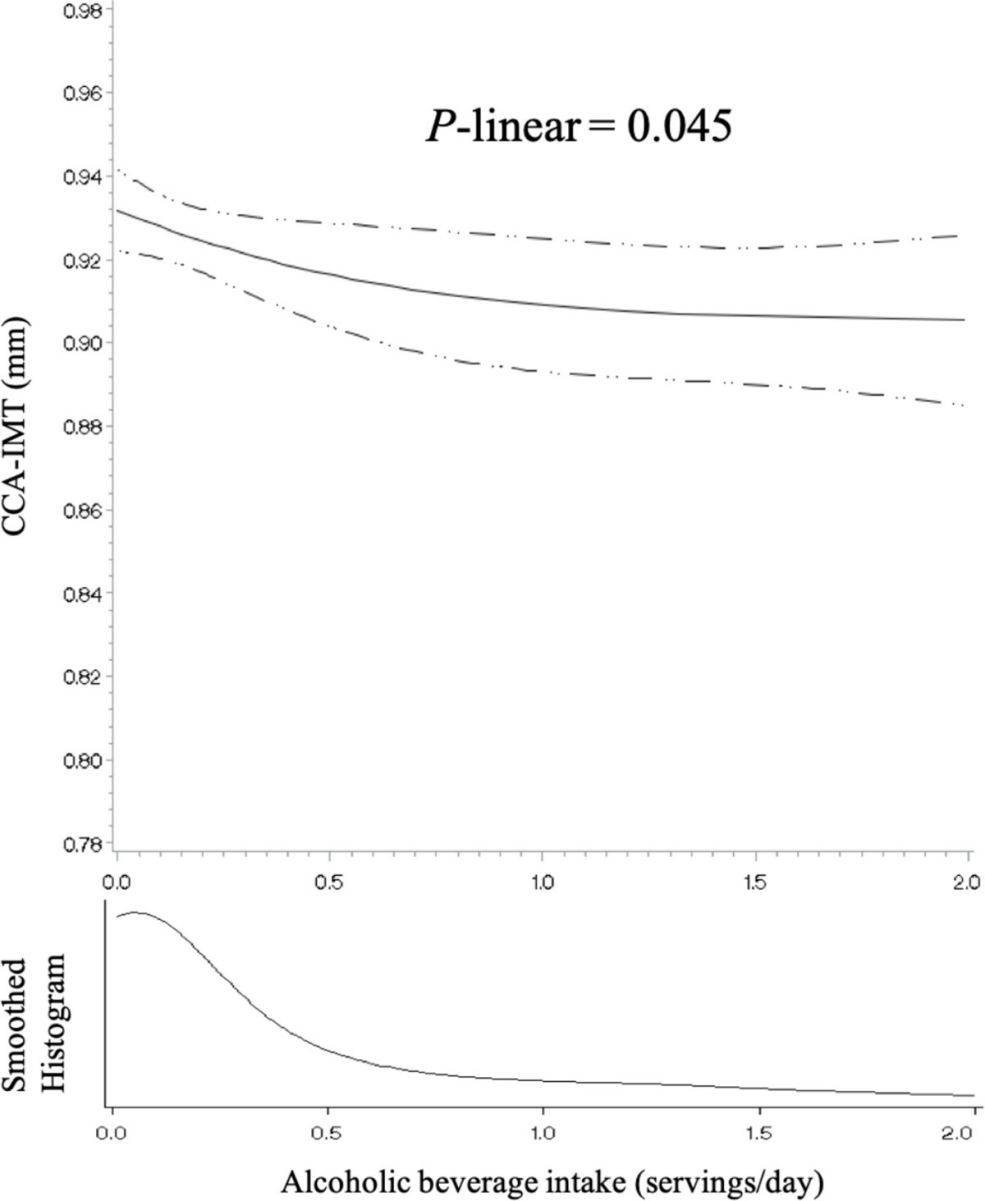
Association between alcoholic beverage intake and common carotid artery intima-media thickness among 1,235 participants of the Study of Women’s Health Across the Nation (United States) using restricted cubic splines, 1996–2013. The solid line represents the predicted least squares means computed using sample mean values for continuous covariates and sample percentages for categorical covariates. The dashed lines represent the 95% confidence limits. Three knots were placed at 5th, 50th, and 95th percentiles of the sample distribution, corresponding to 0, 0.10, and 1.68 servings/d, respectively. One serving of alcoholic beverages was defined as one medium can (355 mL) for beer, one medium glass (148 mL) for wine, and one medium shot (44 mL) for liquor. The model was adjusted for age at the carotid scan, race/ethnicity, education level, financial strain, self-rated overall health, BMI, smoking status, non-occupational physical activity level, menopausal status, use of hormone therapy from baseline to the visit of the carotid scan, the number of missing visits for dietary measurements, total energy intake, Alternate Healthy Eating Index (excluding the alcohol component), coffee intake, elevated blood pressure, elevated fasting glucose, elevated triglycerides, and reduced HDL cholesterol. Extreme alcohol intake (> 2 servings/d) was trimmed from the graph due to sparse data. Abbreviations: CCA, common carotid artery; IMT, intima-media thickness.

The intake of alcoholic beverages was relatively low in the study population. Sixty-two percent of the participants were alcohol drinkers; the average intake among the drinkers was 0.6 serving per day, and only 3% of the participants consumed two or more servings per day. There was an inverse linear association between alcoholic beverage intake and CCA-IMT (Table 3). After adjusting for all covariates, women who consumed > 0 to < 0.5 serving/d, 0.5 to < 1 serving/d, and ≥ 1 serving/d of alcoholic beverages had 0.003 mm (95% CI: -0.019, 0.014), 0.024 mm (95% CI: -0.050, 0.002), and 0.027 mm (95% CI: -0.052, -0.002) smaller CCA-IMT, respectively, than non-drinkers (*P*-trend = 0.014). Restricted cubic spline regression also suggested an inverse linear association (Fig 3). There was no significant association between alcoholic beverage intake and CCA-AD or carotid plaque.

**Table 3:** Average intake of alcoholic beverages and subclinical carotid atherosclerosis among 1,235 participants of the Study of Women’s Health Across the Nation (United States), 1996–2013^a^.

Alcoholic beverages, servings/d					*P*-trend^b^
	None	> 0 to < 0.5	0.5 to < 1	≥ 1	
*n* (%)	472 (38.2)	480 (38.9)	125 (10.1)	158 (12.8)	
CCA-IMT, mm					
Model 1^c^	*Ref*	-0.005 (-0.021, 0.012)	-0.026 (-0.052, -0.000)^f^	-0.028 (-0.053, -0.004)^f^	0.0098
Model 2^d^	*Ref*	-0.004 (-0.020, 0.013)	-0.025 (-0.052, 0.001)	-0.028 (-0.053, -0.003)^f^	0.011
Model 3^e^	*Ref*	-0.003 (-0.019, 0.014)	-0.024 (-0.050, 0.002)	-0.027 (-0.052, -0.002)^f^	0.014
CCA-AD, mm					
Model 1^c^	*Ref*	-0.02 (-0.09, 0.06)	-0.10 (-0.22, 0.02)	-0.05 (-0.17, 0.06)	0.24
Model 2^d^	*Ref*	-0.02 (-0.10, 0.06)	-0.10 (-0.22, 0.03)	-0.05 (-0.17, 0.06)	0.27
Model 3^e^	*Ref*	-0.01 (-0.09, 0.07)	-0.09 (-0.22, 0.03)	-0.06 (-0.18, 0.06)	0.22
Carotid plaque					
Model 1^c^	*Ref*	0.91 (0.73, 1.13)	1.10 (0.79, 1.54)	1.21 (0.90, 1.63)	0.085
Model 2^d^	*Ref*	0.89 (0.71, 1.11)	1.08 (0.77, 1.51)	1.15 (0.85, 1.55)	0.16
Model 3^e^	*Ref*	0.91 (0.73, 1.14)	1.15 (0.82, 1.61)	1.21 (0.90, 1.63)	0.083

Abbreviations: AD, adventitial diameter; CCA, common carotid artery; IMT, intima-media thickness.

^a^ Values for CCA-IMT/CCA-AD are mean differences (95% CIs) from linear models. Values for carotid plaque are risk ratios (95% CIs) of high carotid plaque index (≥ 2) from log-binomial models. Modified Poisson models with robust variance estimation were used to handle model convergence issues. One serving of alcoholic beverages was defined as one medium can (355 mL) for beer, one medium glass (148 mL) for wine, and one medium shot (44 mL) for liquor.

^b^ Computed by assigning the median intake of each category to participants in the corresponding category as a continuous variable.

^c^ Adjusted for age at the carotid scan (continuous), race/ethnicity (African American, Hispanic, Chinese, or non-Hispanic white), education level (≤ high school, some college, or college degree/post-college), financial strain (somewhat/very hard paying for basics, or not hard paying for basics), self-rated overall health (excellent/very good, good, or fair/poor), BMI (continuous), smoking status (never, past, or current), non-occupational physical activity level (continuous), menopausal status (premenopausal or early perimenopausal), use of hormone therapy from baseline to the visit of the carotid scan (ever or never), and the number of missing visits for dietary measurements (0, 1, or 2). The baseline covariates were used unless specified otherwise.

^d^ Model 1 + dietary covariates: The dietary covariates included total energy intake, Alternate Healthy Eating Index (excluding the alcohol component), and intake of coffee, all of which were continuous and the average values across available visits of baseline, Visit 5, and Visit 9.

^e^ Model 2 + cardiovascular risk factors: The cardiovascular risk factors included elevated blood pressure, elevated fasting glucose, elevated triglycerides, and reduced HDL cholesterol, all binary and measured at baseline.

^f^ *P*<0.05 (compared to the reference group).

Women who consumed > 0 to < 0.5 glass/d of whole milk had a 0.043 mm (95% CI: 0.020, 0.065) larger CCA-IMT compared to women who did not drink whole milk, and a 0.062 mm (95% CI: 0.027, 0.097) larger CCA-IMT compared to women who consumed ≥ 0.5 glass/d of whole milk. The difference in CCA-IMT between women who did not drink whole milk and women who drank ≥ 0.5 glass/d was not significant. Women with > 0 to < 0.5 glass/d of whole milk also had a higher risk of carotid plaque than non-drinkers (risk ratio: 1.31; 95% CI: 1.03, 1.67). We found no significant association between whole milk intake and CCA-AD (S2 Table). We did not find significant associations of intakes of tea, SSB, ASB, fruit juices, and milk with lower fat content with measures of subclinical atherosclerosis (S3 to S7 Tables).

The estimates did not change appreciably after accounting for missing data using inverse probability weighting, dropping total energy intake from the full models, or excluding former drinkers. The key findings were qualitatively similar when restricting to the 792 women with beverage data from all three dietary measurements, although many associations became less statistically significant. After excluding the long-term abstainers, there was a nonsignificant inverse linear association between coffee intake and CCA-IMT (*P*-trend = 0.16). The results from the above sensitivity analyses are not shown but are available upon request.

## Discussion

This study evaluated the prospective association between beverage intake during the midlife and subclinical carotid atherosclerosis later in life, measured 14 years after baseline. We found an inverted J-shaped association between coffee intake and CCA-IMT; compared to coffee non-drinkers, women with occasional coffee intake (< 2 cups/d) had a higher CCA-IMT whereas moderate-to-heavy intake (≥ 2 cups/d) was not significantly associated with CCA-IMT. We also found an inverse linear association between alcoholic beverage intake and CCA-IMT, although the study population had few women with very high alcohol intake. Previous work in SWAN has reported that healthy lifestyle and high diet quality are associated with less subclinical atherosclerosis in midlife women [34], while the present study examined how specific beverage intakes may be related to subclinical atherosclerosis.

The benefits and risks of coffee intake have been the subjects of contentious debate for decades. The existing evidence on coffee intake and subclinical atherosclerosis is inconsistent [35–38, 56, 57]. We found an inverted J-shaped association between coffee intake and CCA-IMT, with the largest CCA-IMT observed among occasional drinkers with less than 2 cups of daily coffee intake. After excluding coffee non-drinkers from the analysis, the inverted J-shaped association between coffee intake and CCA-IMT observed in the overall study population was replaced with an inverse linear association, which suggested that the nonlinear association was driven by the small CCA-IMT among long-term coffee abstainers. The cardiovascular impact of coffee is a mix of beneficial and detrimental effects. Antioxidant and anti-inflammatory phenolic compounds in coffee, such as chlorogenic acid, can improve insulin sensitivity and β-cell function [18], prevent oxidation of LDL cholesterol [17], lessen endothelial dysfunction [16], and reduce blood pressure [19], all of which are major contributors to the atherosclerotic process. Further, phytoestrogens in coffee such as lignans may partially replace estrogen after menopause and protect against atherosclerosis [14]. On the other hand, caffeine [13] and chlorogenic acid [15] may elevate the plasma homocysteine level; diterpenoid molecules such as cafestol and kahweol in unfiltered coffee can increase serum total cholesterol levels [58]. The present study suggests that the beneficial properties of the coffee constituents are more likely to offset or outweigh the harmful effects among moderate-to-heavy drinkers compared to among occasional drinkers, in line with the USDA/HHS 2015–2020 *Dietary Guidelines for Americans* which conclude that moderate coffee consumption (3 to 5 cups of coffee per day) is not associated with a higher risk of CVD and can be incorporated into a healthy diet in healthy adults [59]. Given the strong influence of coffee non-drinkers on the results, the findings may also be attributable to residual confounding, as the women who did not drink coffee may have unmeasured characteristics that were not adequately adjusted for in the analyses.

The health implications of alcohol drinking are also among the most controversial issues in nutritional epidemiology. There is a relatively well-documented J-shaped association between alcohol intake and the incidence of clinical CVD, with the lowest risk observed for people who consume one serving of alcoholic beverages per day [25]. Prior studies on the effect of alcohol intake on subclinical atherosclerosis have conflicting results, with some studies reporting a J-shaped association in both women and men [26–28], some finding an association in men but not in women [29, 30], and some failing to detect a meaningful association at all [31–33]. In line with these previous recommendations, we found that the intake of alcoholic beverages was inversely and linearly associated with CCA-IMT within the range of moderate intake (less than two drinks per day). It is worth noting that the potential effect of heavy alcohol intake could not be reliably evaluated in this dissertation as the study population had few women (3%) with more than two servings per day of intake. Therefore, our finding does not contradict previous studies that reported a J-shaped pattern [26–28]. It is likely that alcoholic beverages may be protective against atherosclerosis when consumed in moderation but will have no effect or even detrimental effects when heavily consumed. The USDA/HHS 2015–2020 *Dietary Guidelines for Americans* currently recommends an upper limit of 98 grams per week of alcohol for women, which corresponds to no more than one drink per day [59]. Despite the relatively consistent epidemiologic evidence on the cardiovascular benefit of moderate alcohol intake, public-health guidelines to promote moderate alcohol intake should perhaps not be made universally due to the concern of binge drinking and alcohol abuse. Furthermore, the risks of cancers and diseases of other organs and systems need to be weighed carefully.

Polyphenols in tea, most notably flavonoids, have been shown in cellular and animal studies to have antioxidant, antithrombotic, and anti-inflammatory properties [20–22]. Epidemiologic studies suggest that tea intake may be associated with less atherosclerosis of the aorta and coronary arteries, but the evidence for carotid atherosclerosis is inconsistent [38, 39, 56, 60]. The inconsistency in the literature may be partially attributable to the lower consumption of tea in the U.S. compared to in European countries. The average daily tea intake was approximately 1 cup in this study compared to 2 cups in the Rotterdam Study [39]; 5.5% of the women in the present study consumed ≥ 3 cups/d of tea compared to 12.8% in the French Three-City Study [60]. Overwhelming evidence suggests that the intake of SSB can lead to weight gain, insulin resistance, inflammation, hypertension, visceral adiposity, and atherogenic dyslipidemia [23]. The lack of a clear association for SSB in this study is somewhat surprising and may be due to the low intake and potential reverse causation as women who were aware of higher cardiovascular risk might intentionally reduce their SSB intake. The effect of ASB on atherosclerosis warrants further investigations as ASB may be a reasonable replacement to reduce SSB intake [24]. Evidence on the potential impacts of fruit juices on subclinical atherosclerosis is extremely scarce [61, 62]. Whole milk intake has long been suspected of being a CVD risk factor in adults due to its high saturated fat content and excess calories. Limited prior studies did not find a significant association between milk intake and subclinical atherosclerosis [40, 63]. We do not have a potential etiological explanation for the inverted U-shaped associations of whole milk intake with CCA-IMT and carotid plaque. Only a small proportion (< 20%) of women in our study population were whole milk drinkers, so the unexpected results might be attributable to random error (chance) and certainly need replication.

The three outcomes in this study represent physiologically distinct aspects of the atherosclerotic processes. CCA-IMT is an early marker of carotid atherosclerosis and reflects the thickening of the vessel wall due to lipid deposition, chronic inflammation, infiltration of immunological cells, and hemodynamic changes such as elevated blood pressure [64]. CCA-AD is a marker of vascular remodeling; an elevated CCA-AD represents dilation of the vessel, disturbance in blood flow, and less flexibility to dilate further in response to stimuli [7]. The development of a plaque is the hallmark of atherosclerosis that results from the prolonged accumulation of inflammation, lipid deposition, and calcification [64]. The accumulation of fatty deposits that leads to a thickening of the vessel wall may precede vascular remodeling and plaque formation, which likely explains the lack of association for CCA-AD and carotid plaque. The effect estimates for coffee and alcoholic beverages from the present study may seem small in magnitude. Nevertheless, they are of public-health relevance due to the high prevalence of coffee and alcohol consumption. For example, women with 0.5 to < 1 and ≥ 1 serving/day of alcoholic beverages intake had 17.1% and 19.3% SD smaller CCA-IMT, respectively, than non-drinkers. Every one-SD increment in CCA-IMT has been associated with a 26% higher risk of future myocardial infarction and a 32% higher risk of future stroke [65].

The primary strengths of this study include the focus on midlife women from diverse racial/ethnic backgrounds, the examination of a comprehensive set of beverage groups, and the use of repeated measures of beverage intake. This study also has some limitations. First and foremost, carotid atherosclerosis was measured only once. Without repeated or baseline measures, we were unable to evaluate the change of atherosclerosis over time. As a result, we could not reliably pinpoint the midlife to be the most causally relevant period for the effects of beverage intake. Second, the self-reported beverage intake inevitably had measurement error, which was reduced by using repeated measures from a validated FFQ and by excluding low-quality FFQ data. Third, we could not separately examine the effects of extremely high intakes for some beverages (e.g., > 2 servings/d of alcoholic beverages, > 3 cups/d of tea, or > 2 servings/d of SSB) because such extreme values were rare in the sample. The relatively low variability of some beverages may partially explain the lack of observed associations for those beverages. Fourth, we had no data on coffee brewing methods or decaffeinated coffee intake. Fifth, although we extensively adjusted for covariates, it was not possible to eliminate residual confounding due to the observational nature of the SWAN cohort. Some potential unmeasured confounders include occupation status, socioeconomic position, existing health conditions, and underlying health concerns that influenced the participants’ beverage intakes. Thus, it was not possible to make causal conclusions from this study. Last but not least, due to the large number of models examined, multiple comparisons may be a concern. After the false discovery rate adjustment [66] for the multiple testing of the 24 beverage-outcome combinations, none of the adjusted *P*-trend values remained significant. However, the primary analyses were based on *a priori* hypotheses and have been reported either in the main text or as supplementary tables (S2 to S7 Tables). Still, some of the observed associations might be due to chance and future studies in midlife women are certainly needed to confirm our findings.

In conclusion, this prospective study indicates that occasional coffee intake during the midlife is associated with more subclinical carotid atherosclerosis, whereas moderate-to-heavy coffee intake is not associated with subclinical carotid atherosclerosis later in life. This study also suggests that moderate intake of alcoholic beverages during the midlife is associated with less subclinical carotid atherosclerosis. Future work should focus on the determination of the optimal beverage intake profile for maximum cardiovascular benefits in midlife women.

## Supporting information

S1 Table Beverage items and beverage groups in the Study of Women’s Health Across the Nation (United States), 1996–2013. (DOCX)

S2 Table Average whole milk intake and subclinical carotid atherosclerosis among 1,235 participants of the Study of Women’s Health Across the Nation (United States), 1996–2013. (DOCX)

S3 Table Average tea intake and subclinical carotid atherosclerosis among 1,235 participants of the Study of Women’s Health Across the Nation (United States), 1996–2013. (DOCX)

S4 Table Average sugar-sweetened beverages intake and subclinical carotid atherosclerosis among 1,235 participants of the Study of Women’s Health Across the Nation (United States), 1996–2013. (DOCX)

S5 Table Average artificially sweetened beverages intake and subclinical carotid atherosclerosis among 1,235 participants of the Study of Women’s Health Across the Nation (United States), 1996–2013. (DOCX)

S6 Table Average fruit juices intake and subclinical carotid atherosclerosis among 1,235 participants of the Study of Women’s Health Across the Nation (United States), 1996–2013. (DOCX)

S7 Table Average intake of milk with lower fat content and subclinical carotid atherosclerosis among 1,235 participants of the Study of Women’s Health Across the Nation (United States), 1996–2013. (DOCX)

## References

[1] Benjamin E, Muntner P, Alonso A, Bittencourt M, Callaway C, Carson A, et al Heart Disease and Stroke Statistics—2019 Update. Circulation. 2019;139(10). 10.1161/CIR.0000000000000659 30700139

[2] Shaw LJ, Bugiardini R, Merz CNB. Women and ischemic heart disease: evolving knowledge. Journal of the American College of Cardiology. 2009;54(17):1561–75. 10.1016/j.jacc.2009.04.098 19833255 PMC2789479

[3] Smiath S, Greenland P, Grundy S. Prevention conference V. Beyond secondary prevention: identify the high-risk patient for primary prevention. Executive summary. Circulation. 2000;101:111–6. 10.1161/01.cir.101.1.111 10618313

[4] Stein JH, Korcarz CE, Hurst RT, Lonn E, Kendall CB, Mohler ER, et al Use of carotid ultrasound to identify subclinical vascular disease and evaluate cardiovascular disease risk: a consensus statement from the American Society of Echocardiography Carotid Intima-Media Thickness Task Force endorsed by the Society for Vascular Medicine. Journal of the American Society of Echocardiography. 2008;21(2):93–111. 10.1016/j.echo.2007.11.011 18261694

[5] Mosca L, Benjamin EJ, Berra K, Bezanson JL, Dolor RJ, Lloyd-Jones DM, et al Effectiveness-Based Guidelines for the Prevention of Cardiovascular Disease in Women—2011 Update A Guideline From the American Heart Association. Circulation. 2011;123(11):1243–62. 10.1161/CIR.0b013e31820faaf8 21325087 PMC3182143

[6] Wildman RP, Schott LL, Brockwell S, Kuller LH, Sutton-Tyrrell K. A dietary and exercise intervention slows menopause-associated progression of subclinical atherosclerosis as measured by intima-media thickness of the carotid arteries. Journal of the American College of Cardiology. 2004;44(3):579–85. 10.1016/j.jacc.2004.03.078 15358024

[7] El Khoudary SR, Wildman RP, Matthews K, Thurston RC, Bromberger JT, Sutton-Tyrrell K. Progression rates of carotid intima-media thickness and adventitial diameter during the menopausal transition. Menopause. 2013;20(1):8–14. 10.1097/gme.0b013e3182611787 22990755 PMC3528819

[8] Franklin RM, Ploutz-Snyder L, Kanaley JA. Longitudinal changes in abdominal fat distribution with menopause. Metabolism. 2009;58(3):311–5. 10.1016/j.metabol.2008.09.030 19217444

[9] Greendale GA, Sternfeld B, Huang M, Han W, Karvonen-Gutierrez C, Ruppert K, et al Changes in body composition and weight during the menopause transition. JCI insight. 2019;4(5). 10.1172/jci.insight.124865 PMC6483504 30843880

[10] Matthews KA, Crawford SL, Chae CU, Everson-Rose SA, Sowers MF, Sternfeld B, et al Are changes in cardiovascular disease risk factors in midlife women due to chronological aging or to the menopausal transition? Journal of the American College of Cardiology. 2009;54(25):2366–73. 10.1016/j.jacc.2009.10.009 20082925 PMC2856606

[11] Thurston RC, Karvonen-Gutierrez CA, Derby CA, El Khoudary SR, Kravitz HM, Manson JE. Menopause versus chronologic aging: their roles in women's health. Menopause. 2018;25(8):849–54. 10.1097/GME.0000000000001143 30045364

[12] Wolf A, Bray G, Popkin B. A short history of beverages and how our body treats them. obesity reviews. 2008;9(2):151–64. 10.1111/j.1467-789X.2007.00389.x 18257753

[13] Verhoef P, Pasman WJ, van Vliet T, Urgert R, Katan MB. Contribution of caffeine to the homocysteine-raising effect of coffee: a randomized controlled trial in humans. The American journal of clinical nutrition. 2002;76(6):1244–8. 10.1093/ajcn/76.6.1244 12450889

[14] Peeters P, Grobbee D. Phyto-oestrogens and cardiovascular disease risk. Nutrition, metabolism, and cardiovascular diseases: NMCD. 2000;10(3):154–67. 11006924

[15] Olthof MR, Hollman PC, Zock PL, Katan MB. Consumption of high doses of chlorogenic acid, present in coffee, or of black tea increases plasma total homocysteine concentrations in humans–. The American journal of clinical nutrition. 2001;73(3):532–8. 10.1093/ajcn/73.3.532 11237928

[16] Lopez-Garcia E, van Dam RM, Qi L, Hu FB. Coffee consumption and markers of inflammation and endothelial dysfunction in healthy and diabetic women. The American journal of clinical nutrition. 2006;84(4):888–93. 10.1093/ajcn/84.4.888 17023717

[17] Natella F, Nardini M, Belelli F, Scaccini C. Coffee drinking induces incorporation of phenolic acids into LDL and increases the resistance of LDL to ex vivo oxidation in humans–. The American journal of clinical nutrition. 2007;86(3):604–9. 10.1093/ajcn/86.3.604 17823423

[18] Loopstra-Masters R, Liese A, Haffner S, Wagenknecht L, Hanley A. Associations between the intake of caffeinated and decaffeinated coffee and measures of insulin sensitivity and beta cell function. Diabetologia. 2011;54(2):320–8. 10.1007/s00125-010-1957-8 21046357

[19] Onakpoya I, Spencer E, Thompson M, Heneghan C. The effect of chlorogenic acid on blood pressure: a systematic review and meta-analysis of randomized clinical trials. Journal of human hypertension. 2015;29(2):77 10.1038/jhh.2014.46 24943289

[20] de Whalley CV, Rankin SM, Hoult JRS, Jessup W, Leake DS. Flavonoids inhibit the oxidative modification of low density lipoproteins by macrophages. Biochemical pharmacology. 1990;39(11):1743–50. 10.1016/0006-2952(90)90120-a 2344371

[21] Laughton MJ, Evans PJ, Moroney MA, Hoult J, Halliwell B. Inhibition of mammalian 5-lipoxygenase and cyclo-oxygenase by flavonoids and phenolic dietary additives: relationship to antioxidant activity and to iron ion-reducing ability. Biochemical pharmacology. 1991;42(9):1673–81. 10.1016/0006-2952(91)90501-u 1656994

[22] Kang W-S, Lim I-H, Yuk D-Y, Chung K-H, Park J-B, Yoo H-S, et al Antithrombotic activities of green tea catechins and (−)-epigallocatechin gallate. Thrombosis research. 1999;96(3):229–37. 10588466 10.1016/s0049-3848(99)00104-8

[23] Malik VS, Popkin BM, Bray GA, Després J-P, Hu FB. Sugar-sweetened beverages, obesity, type 2 diabetes mellitus, and cardiovascular disease risk. Circulation. 2010;121(11):1356–64. 10.1161/CIRCULATIONAHA.109.876185 20308626 PMC2862465

[24] Johnson RK, Lichtenstein AH, Anderson CA, Carson JA, Després J-P, Hu FB, et al Low-Calorie Sweetened Beverages and Cardiometabolic Health: A Science Advisory From the American Heart Association. Circulation. 2018;138(9):e126–e40. 10.1161/CIR.0000000000000569 30354445

[25] Ronksley PE, Brien SE, Turner BJ, Mukamal KJ, Ghali WA. Association of alcohol consumption with selected cardiovascular disease outcomes: a systematic review and meta-analysis. Bmj. 2011;342:d671 10.1136/bmj.d671 21343207 PMC3043109

[26] Kiechl S, Willeit J, Rungger G, Egger G, Oberhollenzer F, Bonora E. Alcohol consumption and atherosclerosis: What is the relation? Stroke. 1998;29(5):900–7. 9596232 10.1161/01.str.29.5.900

[27] Mukamal KJ, Kronmal RA, Mittleman MA, O’Leary DH, Polak JF, Cushman M, et al Alcohol consumption and carotid atherosclerosis in older adults. Arteriosclerosis, thrombosis, and vascular biology. 2003;23(12):2252–9. 10.1161/01.ATV.0000101183.58453.39 14563651

[28] Xie X, Ma Y-T, Yang Y-N, Fu Z-Y, Ma X, Huang D, et al Alcohol consumption and carotid atherosclerosis in China: the Cardiovascular Risk Survey. European journal of preventive cardiology. 2012;19(3):314–21. 10.1177/1741826711404501 21450566

[29] Schminke U, Luedemann J, Berger K, Alte D, Mitusch R, Wood WG, et al Association between alcohol consumption and subclinical carotid atherosclerosis. Stroke. 2005;36(8):1746–52. 10.1161/01.STR.0000173159.65228.68 16002763

[30] Zyriax B, Lau K, Klähn T, Boeing H, Völzke H, Windler E. Association between alcohol consumption and carotid intima-media thickness in a healthy population: data of the STRATEGY study (Stress, Atherosclerosis and ECG Study). European journal of clinical nutrition. 2010;64(10):1199 10.1038/ejcn.2010.144 20664623

[31] Demirovic J, Nabulsi A, Folsom AR, Carpenter MA, Szklo M, Sorlie PD, et al Alcohol consumption and ultrasonographically assessed carotid artery wall thickness and distensibility. The Atherosclerosis Risk in Communities (ARIC) Study Investigators. Circulation. 1993;88(6):2787–93. 10.1161/01.cir.88.6.2787 8252692

[32] Tofferi JK, Taylor AJ, Feuerstein IM, O'malley PG. Alcohol intake is not associated with subclinical coronary atherosclerosis. American heart journal. 2004;148(5):803–9. 10.1016/j.ahj.2004.05.023 15523310

[33] Zureik M, Gariépy J, Courbon D, Dartigues J-F, Ritchie K, Tzourio C, et al Alcohol consumption and carotid artery structure in older French adults. Stroke. 2004;35(12):2770–5. 10.1161/01.STR.0000147968.48379.c3 15514169

[34] Wang D, Jackson EA, Karvonen‐Gutierrez CA, Elliott MR, Harlow SD, Hood MM, et al Healthy Lifestyle During the Midlife Is Prospectively Associated With Less Subclinical Carotid Atherosclerosis: The Study of Women's Health Across the Nation. Journal of the American Heart Association. 2018;7(23):e010405 10.1161/JAHA.118.010405 30482079 PMC6405552

[35] van Woudenbergh GJ, Vliegenthart R, van Rooij FJ, Hofman A, Oudkerk M, Witteman JC, et al Coffee consumption and coronary calcification: the Rotterdam Coronary Calcification Study. Arteriosclerosis, thrombosis, and vascular biology. 2008;28(5):1018–23. 10.1161/ATVBAHA.107.160457 18323515

[36] Choi Y, Chang Y, Ryu S, Cho J, Rampal S, Zhang Y, et al Coffee consumption and coronary artery calcium in young and middle-aged asymptomatic adults. Heart. 2015;101(9):686–91. 10.1136/heartjnl-2014-306663 25732752

[37] Patel YR, Gadiraju TV, Ellison RC, Hunt SC, Carr JJ, Heiss G, et al Coffee consumption and calcified atherosclerotic plaques in the coronary arteries: The NHLBI Family Heart Study. Clinical nutrition ESPEN. 2017;17:18–21. 10.1016/j.clnesp.2016.12.003 28361742 PMC5501720

[38] Miller PE, Zhao D, Frazier-Wood AC, Michos ED, Averill M, Sandfort V, et al Associations of Coffee, Tea, and Caffeine Intake with Coronary Artery Calcification and Cardiovascular Events. The American journal of medicine. 2017;130(2):188–97. e5. 10.1016/j.amjmed.2016.08.038 27640739 PMC5263166

[39] Geleijnse JM, Launer LJ, Hofman A, Pols HA, Witteman JC. Tea flavonoids may protect against atherosclerosis: the Rotterdam Study. Archives of Internal Medicine. 1999;159(18):2170–4. 10527294 10.1001/archinte.159.18.2170

[40] Recio-Rodriguez JI, Gomez-Marcos MA, Patino-Alonso M-C, Sanchez A, Agudo-Conde C, Maderuelo-Fernandez JA, et al Association between fat amount of dairy products with pulse wave velocity and carotid intima-media thickness in adults. Nutrition journal. 2014;13(1):37. 24761762 10.1186/1475-2891-13-37 PMC4002866

[41] Sowers MFR, Crawford SL, Sternfeld B, Morganstein D, Gold EB, Greendale GA, et al SWAN: a multicenter, multiethnic, community-based cohort study of women and the menopausal transition In: Lobo RA, Kelsey J, Marcus R, editors. Menopause: Biology and Pathology. San Diego, CA: Academic Press; 2000 p. 175–88.

[42] Ollberding NJ, Wolf RL, Contento I. Food label use and its relation to dietary intake among US adults. Journal of the American Dietetic Association. 2011;111(5):S47–S51. 21515135 10.1016/j.jada.2011.03.009

[43] Block G, Thompson F, Hartman A, Larkin F, Guire K. Comparison of two dietary questionnaires validated against multiple dietary records collected during a 1-year period. Journal of the American Dietetic Association. 1992;92(6):686–93. 1607564

[44] Subar AF, Thompson FE, Kipnis V, Midthune D, Hurwitz P, McNutt S, et al Comparative validation of the Block, Willett, and National Cancer Institute food frequency questionnaires: the Eating at America's Table Study. American journal of epidemiology. 2001;154(12):1089–99. 10.1093/aje/154.12.1089 11744511

[45] Spungen J. Bowes & Church's food values of portions commonly used: Lippincott Williams & Wilkins; 2005.

[46] Huang M-H, Schocken M, Block G, Sowers M, Gold E, Sternfeld B, et al Variation in nutrient intakes by ethnicity: results from the Study of Women's Health Across the Nation (SWAN). Menopause. 2002;9(5):309–19. 12218719 10.1097/00042192-200209000-00003

[47] Thurston RC, Chang Y, Derby CA, Bromberger JT, Harlow SD, Janssen I, et al Abuse and subclinical cardiovascular disease among midlife women. Stroke. 2014;45(8):2246–51. 10.1161/STROKEAHA.114.005928 25034715 PMC4116433

[48] Sutton-Tyrrell K, Kuller LH, Matthews KA, Holubkov R, Patel A, Edmundowicz D, et al Subclinical atherosclerosis in multiple vascular beds: an index of atherosclerotic burden evaluated in postmenopausal women. Atherosclerosis. 2002;160(2):407–16. 11849665 10.1016/s0021-9150(01)00591-3

[49] Matthews KA, El Khoudary SR, Brooks MM, Derby CA, Harlow SD, Barinas-Mitchell EJ, et al Lipid changes around the final menstrual period predict carotid subclinical disease in postmenopausal women. Stroke. 2017;48(1):70–6. 10.1161/STROKEAHA.116.014743 27909203 PMC5183479

[50] Hall MH, Matthews KA, Kravitz HM, Gold EB, Buysse DJ, Bromberger JT, et al Race and financial strain are independent correlates of sleep in midlife women: the SWAN sleep study. Sleep. 2009;32(1):73–82. 19189781 PMC2625326

[51] Sternfeld B, Ainsworth BE, Quesenberry C Jr. Physical activity patterns in a diverse population of women. Preventive medicine. 1999;28(3):313–23. 10.1006/pmed.1998.0470 10072751

[52] McCullough ML, Feskanich D, Stampfer MJ, Giovannucci EL, Rimm EB, Hu FB, et al Diet quality and major chronic disease risk in men and women: moving toward improved dietary guidance. The American journal of clinical nutrition. 2002;76(6):1261–71. 10.1093/ajcn/76.6.1261 12450892

[53] Alberti K, Eckel RH, Grundy SM, Zimmet PZ, Cleeman JI, Donato KA, et al Harmonizing the metabolic syndrome: a joint interim statement of the international diabetes federation task force on epidemiology and prevention; national heart, lung, and blood institute; American heart association; world heart federation; international atherosclerosis society; and international association for the study of obesity. Circulation. 2009;120(16):1640–5. 10.1161/CIRCULATIONAHA.109.192644 19805654

[54] Zou G. A modified poisson regression approach to prospective studies with binary data. American journal of epidemiology. 2004;159(7):702–6. 10.1093/aje/kwh090 15033648

[55] Durrleman S, Simon R. Flexible regression models with cubic splines. Statistics in medicine. 1989;8(5):551–61. 2657958 10.1002/sim.4780080504

[56] Reis JP, Loria CM, Steffen LM, Zhou X, Van Horn L, Siscovick DS, et al Coffee, decaffeinated coffee, caffeine, and tea consumption in young adulthood and atherosclerosis later in life. Arteriosclerosis, thrombosis, and vascular biology. 2010;30(10):2059–66. 10.1161/ATVBAHA.110.208280 20616310 PMC2940975

[57] Miranda AM, Steluti J, Goulart AC, Bensenor IM, Lotufo PA, Marchioni DM. Coffee Consumption and Coronary Artery Calcium Score: Cross‐Sectional Results of ELSA‐Brasil (Brazilian Longitudinal Study of Adult Health). Journal of the American Heart Association. 2018;7(7):e007155 10.1161/JAHA.117.007155 29574458 PMC5907580

[58] Urgert R, Katan M. The cholesterol-raising factor from coffee beans. Annual review of nutrition. 1997;17(1):305–24. 10.1146/annurev.nutr.17.1.305 9240930

[59] 2015–2020 Dietary guidelines for Americans. US Department of Health and Human Services. Washington (DC): USDA2015.

[60] Debette S, Courbon D, Leone N, Gariépy J, Tzourio C, Dartigues J-F, et al Tea consumption is inversely associated with carotid plaques in women. Arteriosclerosis, thrombosis, and vascular biology. 2008;28(2):353–9. 10.1161/ATVBAHA.107.151928 18063810

[61] Davidson MH, Maki KC, Dicklin MR, Feinstein SB, Witchger M, Bell M, et al Effects of consumption of pomegranate juice on carotid intima–media thickness in men and women at moderate risk for coronary heart disease. The American journal of cardiology. 2009;104(7):936–42. 10.1016/j.amjcard.2009.05.037 19766760

[62] Toth PP, Patti AM, Nikolic D, Giglio RV, Castellino G, Biancucci T, et al Bergamot reduces plasma lipids, atherogenic small dense LDL, and subclinical atherosclerosis in subjects with moderate hypercholesterolemia: a 6 months prospective study. Frontiers in pharmacology. 2016;6:299 10.3389/fphar.2015.00299 26779019 PMC4702027

[63] Ivey KL, Lewis JR, Hodgson JM, Zhu K, Dhaliwal SS, Thompson PL, et al Association between yogurt, milk, and cheese consumption and common carotid artery intima-media thickness and cardiovascular disease risk factors in elderly women. The American journal of clinical nutrition. 2011;94(1):234–9. 10.3945/ajcn.111.014159 21613553

[64] Toth P. Subclinical atherosclerosis: what it is, what it means and what we can do about it. International journal of clinical practice. 2008;62(8):1246–54. 10.1111/j.1742-1241.2008.01804.x 18564201 PMC2658007

[65] Lorenz MW, Markus HS, Bots ML, Rosvall M, Sitzer M. Prediction of clinical cardiovascular events with carotid intima-media thickness: a systematic review and meta-analysis. Circulation. 2007;115(4):459–67. 10.1161/CIRCULATIONAHA.106.628875 17242284

[66] Benjamini Y, Hochberg Y. Controlling the false discovery rate: a practical and powerful approach to multiple testing. Journal of the royal statistical society Series B (Methodological). 1995:289–300.

