# Supplementary material for: Prospective associations between beverage intake during the midlife and subclinical carotid atherosclerosis: The Study of Women’s Health Across the Nation
Source: PLoS One. 2019 Jul 10;14(7):e0219301. doi: 10.1371/journal.pone.0219301 (PMC6620009; doi:10.1371/journal.pone.0219301)
Supplement: S1 Table — (DOCX) [file pone.0219301.s001.docx]

#### **S1 Table. Beverage items and beverage groups in the Study of Women’s Health Across the Nation (United States), 1996-2013**

| Beverage item | Beverage group |
| --- | --- |
| Caffeinated coffee | Coffee |
| Green tea | Tea |
| Black tea, English tea, or Chinese tea |  |
| Chinese herbs soup or tea |  |
| Kool-Aid, Hi-C, or other drinks with added vitamin C | Sugar-sweetened beverages |
| Snapple, Calistoga, sweetened bottled waters or iced teas |  |
| Regular cola soft drinks |  |
| Diet cola soft drinks | Artificially sweetened beverages |
| Orange juice or grapefruit juice | Fruit juices |
| Apple juice or grape juice |  |
| Whole milk or whole chocolate milk | Whole milk |
| 2% milk or 2% chocolate milk | Milk with lower fat content |
| Skim milk or 1% milk |  |
| Beer | Alcoholic beverages |
| Wine or wine coolers |  |
| Liquor or mixed drinks |  |
| Cream, half and half, or nondairy creamer in coffee or tea | Beverage condiments |
| Milk in coffee or tea |  |
| Sugar or honey in coffee or tea or on cereal |  |

Beverage item soy milk was not considered in the analysis due to low intake and incompatibility with other beverage groups.
