# Supplementary material for: Prospective associations between beverage intake during the midlife and subclinical carotid atherosclerosis: The Study of Women’s Health Across the Nation
Source: PLoS One. 2019 Jul 10;14(7):e0219301. doi: 10.1371/journal.pone.0219301 (PMC6620009; doi:10.1371/journal.pone.0219301)
Supplement: S4 Table — (DOCX) [file pone.0219301.s004.docx]

**S4 Table. Average sugar-sweetened beverages intake and subclinical carotid atherosclerosis among 1,235 participants of the Study of Women’s Health Across the Nation (United States), 1996-2013^a^**

| SSB, servings/d |  |  |  |  | *P*-trend^b^ |
| --- | --- | --- | --- | --- | --- |
|  | None | > 0 to < 0.5 | 0.5 to < 1 | ≥ 1 |  |
| *n* (%) | 227 (18.4) | 598 (48.4) | 185 (15.0) | 225 (18.2) |  |
| CCA-IMT, mm |  |  |  |  |  |
| Model 1^c^ | *Ref* | 0.014 (-0.006, 0.034) | 0.003 (-0.023, 0.029) | 0.029 (0.004, 0.054)^f^ | 0.076 |
| Model 2^d^ | *Ref* | 0.013 (-0.007, 0.033) | 0.001 (-0.024, 0.027) | 0.027 (0.002, 0.053)^f^ | 0.11 |
| Model 3^e^ | *Ref* | 0.013 (-0.007, 0.032) | -0.003 (-0.028, 0.023) | 0.017 (-0.009, 0.043) | 0.52 |
| CCA-AD, mm |  |  |  |  |  |
| Model 1^c^ | *Ref* | 0.01 (-0.09, 0.10) | 0.03 (-0.10, 0.15) | 0.10 (-0.02, 0.22) | 0.061 |
| Model 2^d^ | *Ref* | 0.00 (-0.10, 0.09) | 0.01 (-0.11, 0.14) | 0.08 (-0.05, 0.20) | 0.12 |
| Model 3^e^ | *Ref* | -0.01 (-0.10, 0.08) | -0.01 (-0.13, 0.11) | 0.02 (-0.10, 0.15) | 0.59 |
| Carotid plaque |  |  |  |  |  |
| Model 1^c^ | *Ref* | 1.28 (0.97, 1.68) | 1.29 (0.91, 1.83) | 1.28 (0.92, 1.78) | 0.49 |
| Model 2^d^ | *Ref* | 1.27 (0.97, 1.67) | 1.29 (0.90, 1.83) | 1.27 (0.91, 1.78) | 0.53 |
| Model 3^e^ | *Ref* | 1.28 (0.98, 1.68) | 1.19 (0.84, 1.70) | 1.11 (0.79, 1.55) | 0.60 |

Abbreviations: AD, adventitial diameter; CCA, common carotid artery; IMT, intima-media thickness; SSB, sugar-sweetened beverages.

^a^ Values for CCA-IMT/CCA-AD are mean differences (95% CIs)from linear models. Values for carotid plaque are risk ratios (95% CIs) of high carotid plaque index (≥ 2) from log-binomial models. Modified Poisson models with robust variance estimation were used to handle model convergence issues. One serving of SSB was defined as one medium can (355 mL).

^b^ Computed by assigning the median intake of each category to participants in the corresponding category as a continuous variable.

^c^ Adjusted for age at the carotid scan (continuous), race/ethnicity (African American, Hispanic, Chinese, or non-Hispanic white), education level (≤ high school, some college, or college degree/post-college), financial strain (somewhat/very hard paying for basics, or not hard paying for basics), self-rated overall health (excellent/very good, good, or fair/poor), smoking status (never, past, or current), non-occupational physical activity level (continuous), menopausal status (premenopausal or early perimenopausal), use of hormone therapy from baseline to the visit of the carotid scan (ever or never), and the number of missing visits for dietary measurements (0, 1, or 2). The baseline covariates were used unless specified otherwise

^d^ Model 1 + dietary covariates: The dietary covariates included Alternate Healthy Eating Index and intake of fruit juices, both of which were continuous and the average values across available visits of baseline, Visit 5, and Visit 9.

^e^ Model 2 + cardiovascular risk factors: The cardiovascular risk factors included BMI, total energy intake, elevated blood pressure, elevated fasting glucose, elevated triglycerides, and reduced HDL cholesterol. BMI was the continuous value at baseline. Total energy intake was the continuous average value across available visits of baseline, Visit 5, and Visit 9. The other four covariates were all binary and measured at baseline.

^f^ *P*<0.05 (compared to the reference group).
